# Failure to Affect Decision Criteria During Recognition Memory With Continuous Theta Burst Stimulation

**DOI:** 10.3389/fnins.2018.00705

**Published:** 2018-10-11

**Authors:** Evan Layher, Tyler Santander, Lukas J. Volz, Michael B. Miller

**Affiliations:** ^1^Department of Psychological and Brain Sciences, University of California, Santa Barbara, Santa Barbara, CA, United States; ^2^Department of Neurology, University Hospital Cologne, Cologne, Germany; ^3^SAGE Center for the Study of the Mind, University of California, Santa Barbara, Santa Barbara, CA, United States

**Keywords:** continuous theta burst stimulation (cTBS), recognition memory, criterion shifting, functional magnetic brain imaging (fMRI), prefrontal cortex

## Abstract

A decision criterion establishes the minimum amount of memory evidence required for recognition. When a liberal criterion is set, items are recognized based on weak evidence whereas a conservative criterion requires greater memory strength for recognition. The decision criterion is a fundamental aspect of recognition memory but little is known about the underlying neural mechanisms of maintaining a criterion. We used continuous theta burst stimulation (cTBS) with the goal of inhibiting prefrontal cortex excitability while participants performed recognition tests. We hypothesized that inhibiting the right inferior frontal gyrus (rIFG), right middle frontal gyrus (rMFG), and right dorsolateral prefrontal cortex (rDLPFC) would cause participants to establish less conservative decision criteria without affecting recognition memory performance. Participants initially performed recognition memory tests while maintaining conservative decision criteria during fMRI scanning. Peak activity in the successful retrieval effect contrast (Hits > Correct Rejections) provided subject-specific cTBS target sites. During three separate sessions, participants completed the same recognition memory paradigm while maintaining conservative and liberal decision criteria both before and after cTBS. Across two experiments we failed to significantly alter decision criteria placement by applying cTBS to the rIFG, rMFG, and rDLPFC despite efforts to precisely target individualized brain areas. However, we unexpectedly improved discriminability following cTBS to the rDLPFC specifically when participants maintained a liberal criterion. Although this finding may guide future studies investigating the neural mechanisms underlying discriminability in recognition memory, cTBS proved ineffective at altering decision criteria.

## Introduction

When making a recognition memory judgment, individuals must compare the strength of memory evidence elicited by an item to a decision criterion. If the memory strength exceeds the decision threshold, then one will indicate that he or she recognizes the item—otherwise the item is considered novel. When items are recognized based on weak memory evidence, a liberal decision criterion is employed. Conversely, a conservative decision criterion is established when items require strong memory evidence for recognition. The appropriate placement of a decision criterion can improve the outcomes of memory-based decisions. For instance, a guard at a security checkpoint should establish a liberal criterion by stopping and questioning people who vaguely resemble a known criminal because questioning an innocent person is only a minor inconvenience. However, when the same guard is faced with the same memory evidence in situations that may require physical force, a conservative criterion should be established to avoid harming innocent people. Despite the importance of establishing a decision criterion based on memory evidence, little is known about how these criteria are set and the neural mechanisms that underlie them (Gold and Shadlen, 2007; Ratcliff et al., 2016).

There is evidence to suggest the prefrontal cortex plays a role in maintaining a conservative decision criterion. Patients with frontal lobe lesions tend to establish more liberal decision criteria as evidenced by increased false alarm rates during recognition memory (Parkin et al., 1996; Schacter et al., 1996; Swick and Knight, 1999; Verfaellie et al., 2004; Callahan et al., 2011; Biesbroek et al., 2015). A tendency to set liberal decision criteria is also observed in other patient populations associated with frontal lobe damage or dysfunction, including Alzheimer's disease (Budson et al., 2006; Waring et al., 2008; Beth et al., 2009; Deason et al., 2017) and schizophrenia (Moritz et al., 2008). Prefrontal cortex processes can also be disrupted through drug administration, such as with **Δ**9-tetrahydrocannabinol (THC) (Bossong et al., 2012), which demonstrated increased false alarm rates during recognition memory (Doss et al., 2018). Taken together, these studies strongly suggest that a dysfunctional prefrontal cortex impairs the ability to set conservative criteria.

Research in healthy individuals also supports the notion that maintaining a conservative criterion during recognition memory requires engagement of the prefrontal cortex. In particular, Aminoff et al. (2015) sought to manipulate criterion placement as participants performed recognition memory tests during fMRI scanning. An investigation of the successful retrieval effect, which contrasts hit trials against correct rejection trials (H > CR), yielded robust recruitment of widespread fronto-parietal regions when participants maintained a conservative criterion—but not when maintaining a liberal criterion. These findings directly oppose hypotheses that attribute increased BOLD activity in the H > CR contrast to differences in memory strength, since hit trials (on average) confer stronger memory evidence relative to correct rejection trials (Wheeler and Buckner, 2003; Kahn et al., 2004; Wagner et al., 2005; Vilberg and Rugg, 2009; Yu et al., 2012; Criss et al., 2013). However, the H > CR contrast also carries information about memory-based decisional processes: hit responses represent a *decision* that the memory evidence of an item exceeds the established criterion, whereas correctly rejected items do not carry enough memory evidence to surpass the decision threshold (O'Connor et al., 2010; Jaegar et al., 2013; Miller and Dobbins, 2014). Through an individual differences analysis, Aminoff et al. (2015) revealed that the more conservatively a participant responded the greater the fronto-parietal activity in the H > CR contrast. No such relationship existed between fronto-parietal activity and individual differences in memory strength. This finding provides compelling evidence that the observed fronto-parietal activity in the H > CR contrast is not only associated with the maintenance of a conservative criterion, but that the *magnitude* of the fronto-parietal activity correlates with the conservativeness of a decision criterion.

One potential explanation for the robust activity in the H > CR contrast when a conservative criterion is maintained is that suppressing a prepotent familiarity response may require cognitive control processes related to response inhibition (see Aminoff et al., 2015). In particular, there is strong evidence indicating that the right inferior frontal gyrus (rIFG) is implicated in response inhibition (Wager et al., 2005; Chambers et al., 2009; Bari and Robbins, 2013) and may serve as a cognitive braking system (Aron et al., 2014, 2015). Other prefrontal areas, such as the dorsolateral prefrontal cortex (DLPFC), may also play a role in maintaining task goals to prepare for inhibiting a response (Jahfari et al., 2010; Swann et al., 2013). If maintaining a conservative criterion requires preparing for or executing response inhibition, then the rIFG and surrounding prefrontal areas provide promising sites for further investigation.

Functional MRI studies are of course limited in their ability to draw *causal* inferences between brain activity and behavior. However, the advent of neurostimulation techniques, such as repetitive transcranial magnetic stimulation (rTMS), offers a direct means of testing whether overt behavior can be altered by targeted cortical stimulation. Previous rTMS research demonstrated that offline continuous theta burst stimulation (cTBS) serves as an effective inhibitor of cortical excitability for up to 60 min in the hand area of the motor cortex (Huang et al., 2005). Although it is unclear whether offline cTBS has equivalent inhibitory effects when applied to areas within the prefrontal cortex (Grossheinrich et al., 2009), a handful of studies have successfully manipulated cognitive performance by applying offline cTBS to prefrontal regions. For example, Verbruggen et al. (2010) disrupted response inhibition and dual-task performance after applying cTBS to the rIFG. Georgiev et al. (2016) applied cTBS over the rDLPFC, which led to slower response times during a perceptual decision-making task. Additionally, Cho et al. (2010) reduced impulsivity in a delayed discounting task after applying cTBS to the rDLPFC. These studies provide evidence that offline cTBS can affect decision-making performance in a seemingly inhibitory manner.

Given that cTBS appears to inhibit prefrontal cortex excitability, we attempted to causally manipulate criterion placement by applying cTBS to brain regions that Aminoff et al. (2015) identified as being associated with the magnitude of a conservative decision criterion—namely, the rIFG, rMFG, and rDLPFC. We hypothesized that cTBS to the rIFG, rMFG, and rDLPFC would inhibit the function of networks implicated in criterion placement without affecting recognition memory accuracy. More specifically, we predicted that individuals would establish less conservative criteria when a conservative criterion is advantageous. In situations where a liberal criterion is advantageous, we expected no changes in criterion placement. This finding would suggest that the rIFG, rMFG, and rDLPFC play a crucial role in maintaining conservative decision criteria but are non-essential for maintaining liberal decision criteria during recognition memory. Importantly, this approach can provide more concrete evidence to support previous observations of increased fronto-parietal activity during successful retrieval—but only when a conservative criterion is maintained (e.g., Aminoff et al., 2015)—and help explain why individuals with damaged and/or dysfunctional prefrontal cortices generally set liberal decision criteria relative to healthy controls.

## Materials and methods

### Participants

Prior to the cTBS experiment, 352 participants (126 males; aged 18–38; *M* = 20.1 ± 2.5 *SD*) conducted the initial prescreen task. Participants received an invitation to partake in the neuroimaging and neurostimulation phases of the experiment if they discriminated between old and new images above chance, sufficiently shifted criteria between the conservative and liberal conditions, and met all of the eligibility requirements for MRI and TMS (See Procedure). 20 participants did not receive an invitation due to below chance discriminability performance; an additional 150 participants did not receive an invitation because they did not adequately shift between conservative and liberal decision criteria. The 182 eligible participants received an invitation to participate in the study on a rolling basis; enrollment consisted of participants who replied quickest to the invitation.

Ultimately, a total of 36 participants (9 males; aged 18-26; *M* = 20.0 ± 1.7 *SD*) successfully completed all three cTBS sessions between Experiment 1 and Experiment 2. The first experiment consisted of 20 participants (5 males; aged 18–23; *M* = 19.7 ± 1.6 *SD*) with the exclusion of four additional participants due to computer malfunction (1), procedural error (1), or incomplete stimulation (2) during at least one of the three cTBS sessions. After observing a surprising trend (see Results) we conducted a follow-up in Experiment 2 with 16 participants (4 males; aged 19–26; *M* = 20.5 ± 1.8 *SD*). Two additional participants withdrew from the second experiment.

Participants enrolled in the study via the University of California Santa Barbara (UCSB) paid research participation website. Participants received $10/h for performing the prescreen task and $20/h for conducting the MRI and cTBS sessions. The study received approval from the UCSB Human Subjects Committee Institutional Review Board and all subjects gave written informed consent.

### Procedure

Both Experiment 1 and Experiment 2 consisted of a prescreen recognition memory task, an fMRI scanning session, and three cTBS sessions. The prescreen recognition memory task identified participants that discriminated between studied and unstudied face stimuli and adaptively shifted between conservative and liberal decision criteria. We intentionally made discriminability difficult to motivate subjects to bias his or her responses, but required above chance discriminability performance to ensure participants correctly conducted the recognition memory task.

In addition to performing above chance on the recognition memory task, participants also needed to adaptively shift their decision criteria. There are vast individual differences in the placement of a decision criterion during a recognition memory test (Aminoff et al., 2012; Kantner and Lindsay, 2012, 2014; Kantner et al., 2015; Frithsen et al., 2017). Therefore, we needed to identify when participants establish a conservative decision criterion *relative* to a more liberal decision criterion. Additionally, Aminoff et al. (2015) found that individuals who failed to shift their decision criteria did not exhibit robust fronto-parietal activity in the H > CR contrast, even in situations where maintaining a conservative criterion is advantageous. To test whether cTBS disrupts maintaining a *relatively* more conservative decision criterion and to ensure we obtain robust fMRI activation in the H > CR contrast for precise individualized cTBS targeting, we only invited individuals to participate in the study if they adaptively shifted criteria during the initial prescreen recognition memory tests. Once selected, participants performed recognition memory tests while maintaining a conservative decision criterion during fMRI scanning. The fMRI analyses provided subject-specific cTBS target sites based on each participant's peak voxel activity in the H > CR contrast within the rIFG (Experiments 1 and 2), rMFG (Experiment 1), and rDLPFC (Experiment 2). Finally, participants conducted recognition memory tests both before and after cTBS on three separate visits.

### Recognition memory task

**Figure 1:**
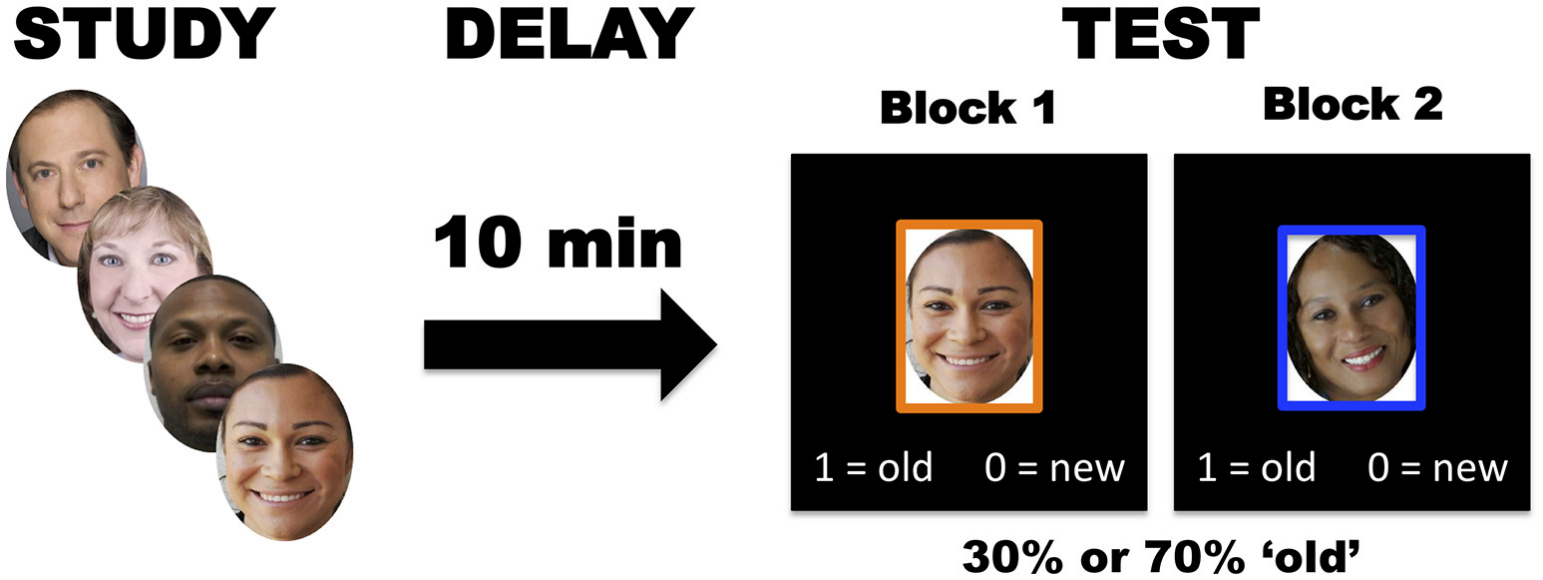
In the recognition memory task, participants studied 100 face images, followed by liberal and conservative test blocks (100 trials per block) after a 10-minute delay.

The recognition memory task followed the same procedure for the initial prescreen, fMRI, and cTBS phases of the experiment unless otherwise specified (Figure 1). During the study session, participants passively viewed a series of 100 novel face images displayed in the center of a computer screen with a black background. Each study image appeared rapidly for 300 ms followed by a 200 ms blank screen interstimulus interval to intentionally induce low discrimination levels, thus making criterion shifting more advantageous. Every participant viewed a random series of images drawn from the 10k US Adult Faces database (Bainbridge et al., 2013) and images did not repeat across sessions.

After the study session, participants completed a test session that consisted of two test blocks in which participants made “old” (previously studied) or “new” (unstudied) recognition judgments. Prior to each test block, explicit instructions informed participants of the base rate probabilities of encountering a previously studied item. In the low probability (conservative criterion) condition, only 30% of the test items appeared during the study phase, making it advantageous to respond “new” more often. In the high probability (liberal criterion) condition, 70% of test items appeared during the study phase making “old” responses more advantageous. Each block consisted of 100 test trials: conservative test blocks contained 30 old images and 70 new images, while liberal test blocks contained 70 old images and 30 new images. Every test image appeared in the center of a computer screen surrounded by an orange or blue frame to remind participants of the relevant test contingencies (conservative or liberal criterion). The images remained on screen until the participant made a response. During each trial, instructions appeared at the bottom of the screen to indicate whether the “0” or “1” keyboard button represented an “old” or “new” response. The order of test liberal/conservative test blocks, frame color associations, and keyboard assignments were randomized across participants. In total, the recognition memory task spanned 10 to 15 min. Administration of the task occurred within MATLAB version R2016B and incorporated open source code from Psychophysics Toolbox, v3 (Brainard, 1997).

### Signal detection theory

We used signal detection theory (SDT) to assess recognition memory performance. For each test block, we computed hit rates (number of items correctly identified as “old” relative to the total number of old stimuli) and false alarm rates (number of items incorrectly called “old” relative to the number of new stimuli) and obtained measures of discriminability (*d'*), criterion placement (*c*), and criterion shifting (*C*) through the following equations (Stanislaw and Todorov, 1999; Macmillan and Creelman, 2005):

$$\begin{array}{lll} \text{d'} & = & \text{z}\left(\text{hit rate}\right)-\text{z}\left(\text{false alarm rate}\right)\\ \text{c} & = & -0.5^{*}\left[\text{z}\left(\text{hit rate}\right)+\text{z}\left(\text{false alarm rate}\right)\right]\\ \text{C} & = & {\text{c}}_{\left(\text{conservative}\right)}-{\text{c}}_{\left(\text{liberal}\right)}, \end{array}$$

where *z* gives the density of the standard normal distribution. In the three instances that a participant attained a false alarm rate of 0% an addition of 0.5 to the numerator of the rate prevented an infinite normalized value (see Macmillan and Kaplan, 1985).

Although criterion placement and discriminability are behaviorally-independent processes, there is a statistical relationship between the optimal criterion placement and the extent of discriminability (Macmillan and Creelman, 2005). In other words, as discriminability increases, the optimal criterion placement for a recognition memory test (with a biased probability) will approach 0. To control for the influence of discriminability on criterion placement, we residualized *c* against *d'* across all participants within each cTBS session. This yielded normalized *c* values to ensure statistical independence between *c* and *d'* (see Aminoff et al., 2012). All subsequent analyses used the normalized *c* values to assess criterion placement and shifting.

### Deriving subject-specific cTBS targets

Participants selected from the prescreen initially conducted a modified version of the recognition memory task during fMRI scanning. In this version, participants studied 60 face images and performed two conservative testing blocks—both to precisely identify regions supporting conservative criterion placement, and because the H > CR contrast does not reveal robust activity when a liberal criterion is set (Aminoff et al., 2015). Each test block contained 100 test trials with 30 old and 70 new images that appeared for 3 s with random jitter to ensure separability of hemodynamic responses (interstimulus intervals of 0–6 s). Participants responded via a two-button response box held in the right hand.

A Siemens 3T PRISMA MRI scanner collected all imaging data using a 64 channel head and neck coil. An initial magnetization-prepared rapid gradient echo (MPRAGE) sequence acquired *T*_1_-weighted anatomical images (208 slices; TE = 2.22 ms; TR = 2,500 ms; FoV = 241 mm^2^; voxel size: 0.9 mm^3^). A subsequent $T_{2}^{*}$-weighted gradient recall echo (GRE) field map scan (48 oblique slices; TE_1_ = 4.92 ms; TE_2_ = 7.38 ms; FoV = 192 mm^2^; voxel size: 3 mm^3^) provided estimates of magnetic field inhomogeneities. Functional image acquisition employed a ${}_{2}^{*}$-weighted multi-band echo planar imaging (mbEPI) sequence sensitive to the BOLD contrast (48 oblique slices; TE = 35 ms; TR = 400 ms; FoV = 192 mm^2^; voxel size: 3 mm^3^; multiband factor = 8). Total scanning time lasted approximately 30 min.

All fMRI preprocessing and statistical analyses occurred using the FMRI Brain Software Library (FSL), v5.0 (Jenkinson et al., 2012). Each functional scan underwent motion correction and realignment to the middle volume using FSL MCFLIRT. FSL FUGUE unwarped geometric deformations due to motion and field inhomogeneities. Temporal preprocessing of voxelwise timeseries included both high pass filtering (0.01 Hz) and prewhitening. The data underwent spatial smoothing using a 5 mm^3^ full-width at half-maximum (FWHM) Gaussian kernel. Coregistration of functional data to each individual's *T*_1_-weighted anatomical image enabled cTBS target identification in subject space.

An event-related general linear model (GLM) identified within-subject activity related to successful retrieval. Each test block contained 4 regressors of interest: hits, correct rejections, misses, and false alarms. Nuisance regressors included trials with no old/new response in addition to head motion parameters derived from MCFLIRT realignment. FSL FEAT provided model estimates to compute H > CR contrasts for each individual.

The H > CR contrast provided subject-specific cTBS target sites based on the peak voxel within each ROI. We anatomically-defined rIFG and rMFG ROIs according to the Harvard-Oxford Cortical Structural atlas in FSL, using probability maps with a threshold of 30% for the combined right pars opercularis and pars triangularis maps (rIFG) and the rMFG map. For Experiment 1, sought to ensure that individual variation in the location of the peak voxel within the H > CR contrast would be encompassed by our large anatomical ROIs; in Experiment 2, we functionally defined the rDLPFC ROI from the Aminoff et al. (2015) group-level H > CR contrast (see Results), specifically for the conservative criterion condition of the recognition memory test for faces. FSL FLIRT registered each ROI to a participant's native brain space.

To ensure replication of the fMRI findings from Aminoff et al. (2015), we performed a group-level mixed-effects analysis of variance for the H > CR contrast. The resulting *Z* statistic maps underwent whole-brain voxelwise thresholding at *Z* > 3.1 and cluster correction (*p* < 0.05) using Gaussian random field theory.

### cTBS

Participants attended three cTBS sessions each separated by at least 48 h to ensure that the effects of stimulation from one session did not carry over to another session. The location of the target site differed for each of the three cTBS sessions. In the first experiment participants received cTBS to the rIFG, rMFG, or occipital vertex (sham stimulation). The second experiment followed the same procedures except participants received cTBS to the rDLPFC instead of the rMFG (see Results). The order of stimulation over the three sessions occurred pseudo-randomly across participants to include all six possible order combinations. The cTBS stimulation intensity remained fixed at 35% of the maximum stimulator output because rIFG stimulation inadvertently contracts the temporalis muscle and the chosen intensity level minimized discomfort. During cTBS to the rMFG and rDLPFC a researcher held the TMS coil handle at a 45° angle relative to the head's midline with the handle pointing posteriorly and to the right. The TMS coil handle pointed posteriorly while aligned parallel to the head's midline during cTBS to the rIFG and occipital vertex. Participants unknowingly received sham stimulation that involved a slight tilting of the TMS coil away from the scalp to mitigate cTBS effects on the occipital vertex.

To precisely stimulate the functionally defined cTBS target sites, participants wore a headband with an infrared tracking device and earplugs to protect against hearing loss from the ambient TMS noise. A pointer tool registered the position of the tracking device in the participant's headband to the participant's *T*_1_-weighted anatomical image using a Polaris infrared optical tracking system (Northern Digital Inc., Waterloo, ON, Canada) in conjunction with the Brainsight TMS navigation system, v2.3.9 (Rogue Research, Montreal, QC, Canada). This allowed for real-time tracking of the position of the TMS coil relative to the cTBS target sites within each participant's brain leading to precise stimulation of the target site. A 70 mm figure of eight coil delivered cTBS in bursts of 50 Hz triplets at a rate of 5 Hz for 40 s (600 total pulses) using a Magstim Rapid^2^ stimulator unit (Magstim Inc., Morrisville, NC).

**Figure 2:**
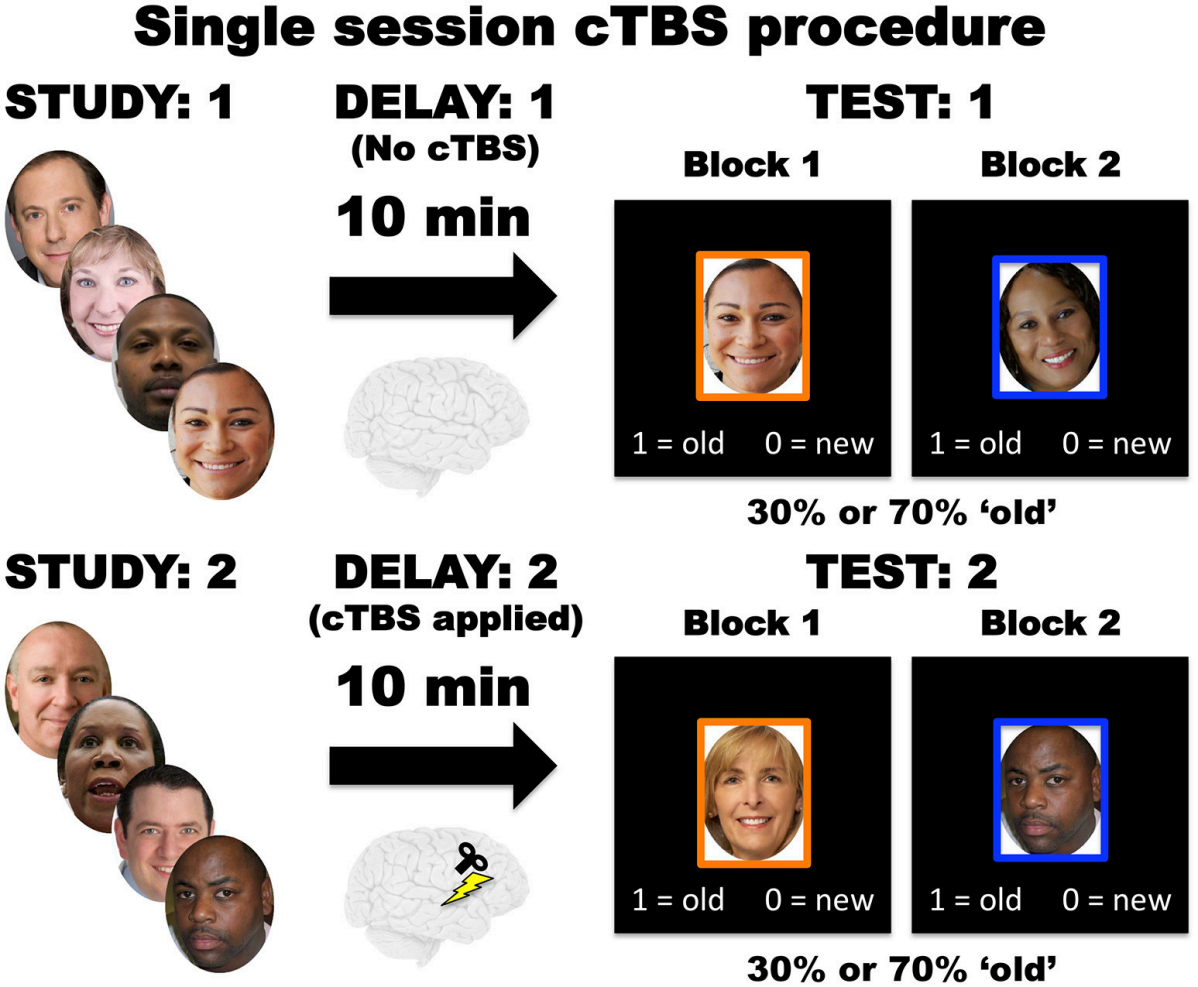
During each of the three cTBS sessions, participants initially conducted the recognition memory task without cTBS stimulation. Then participants performed the recognition memory task again with cTBS applied during the delay period.

During each of the three cTBS session, participants performed the recognition memory task twice, once before cTBS and again after stimulation. Following the first study phase, a 10-min delay ensued where participants sat in a chair while researchers provided information about the cTBS procedure for that session. Participants then completed the first test phase and immediately began the study phase of the second run. After the second study phase, researchers applied cTBS during another 10-min delay period. Afterwards, participants performed the second recognition memory test phase (Figure 2).

### Statistical analysis

We tested the effects of cTBS stimulation on criterion placement and discriminability using linear mixed models, implemented with the lme4 package (Bates et al., 2015) in R. In Experiment 1, deviation contrasts specified fixed effects, which modeled mean differences in *d'* and normalized *c* as functions of criterion condition (liberal > conservative), task time (post- > pre-cTBS), and cTBS target site (rIFG > sham and rMFG > sham). We additionally modeled three-way interactions between criterion, time, and cTBS target contrasts, along with all marginal two-way interactions. Thus, the fixed effects models took the following form:

$$\begin{array}{l} ŷ=b_{0}+b_{1}\left(lib>con\right)+b_{2}\left(post>pre\right)+b_{3}\left(rIFG>sham\right)\\ \;+b_{4}\left(rMFG>sham\right)+b_{5}\left(lib>con\;*\;post>pre\right)\\ \;+b_{6}\left(lib>con\;*\;rIFG>sham\right)+b_{7}\left(lib>con\;*\;rMFG>sham\right)\\ \;+b_{8}\left(post>pre\;*\;rIFG>sham\right)+b_{9}\left(post>pre\;*\;rMFG>sham\right)\\ \;+b_{10}\left(lib>con\;*\;post>pre\;*\;rIFG>sham\right)\\ \;+b_{11}\left(lib>con\;*\;post>pre\;*\;rMFG>sham\right)+\varepsilon . \end{array}$$

The Experiment 2 models remained identical in form with the substitution of rDLPFC for rMFG. In all cases, we specified a random effect on the model intercept across subjects to account for baseline variation in *c* and *d*'.

Linear mixed models do not yield *p*-values for parameter estimates due to inherent difficulties in estimating denominator degrees of freedom. However, the restricted maximum likelihood approach to model estimation yields a posterior distribution over the parameters, allowing us to construct empirical confidence intervals via simulation. We performed 1,000 iterations of posterior simulation to approximate 95% CIs around each parameter estimate. Any CI spanning zero is considered non-significant. We also report effect size approximations of Cohen's *d*, obtained by dividing contrast parameter estimates by the square root of the total random effects variance of the model (Westfall et al., 2014).

## Results

### The successful retrieval effect

**Figure 3:**
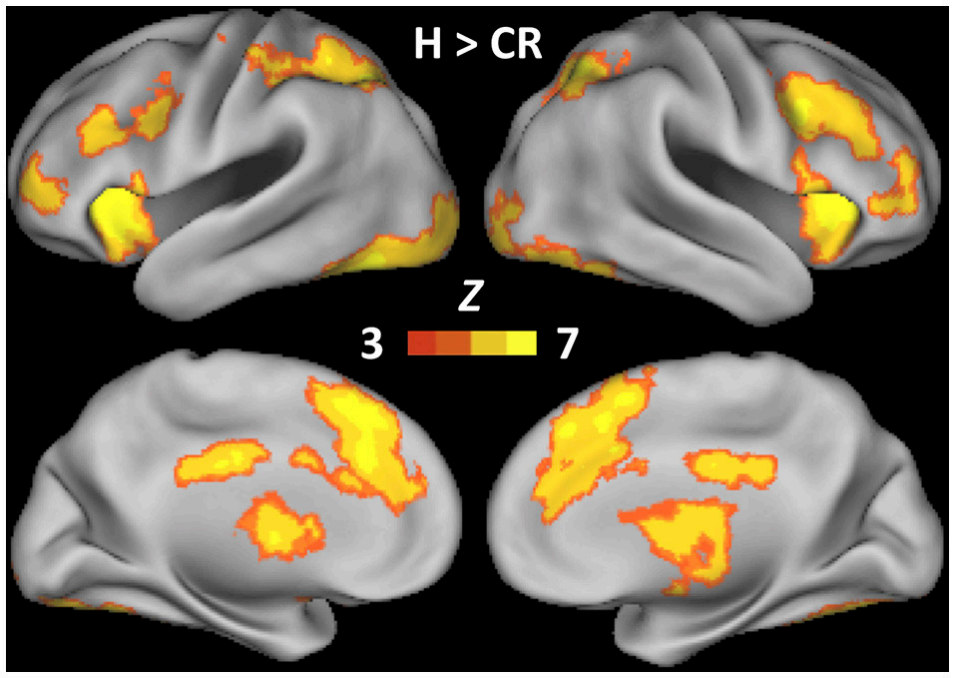
Whole-brain statistical Z-map of Hits > Correct Rejections, estimated over two recognition memory tests requiring participants to maintain a conservative decision criterion (*N* = 36). Thresholding at Z > 3.1 and cluster correction (*p* = 0.05) determined significance.

**Figure 4:**
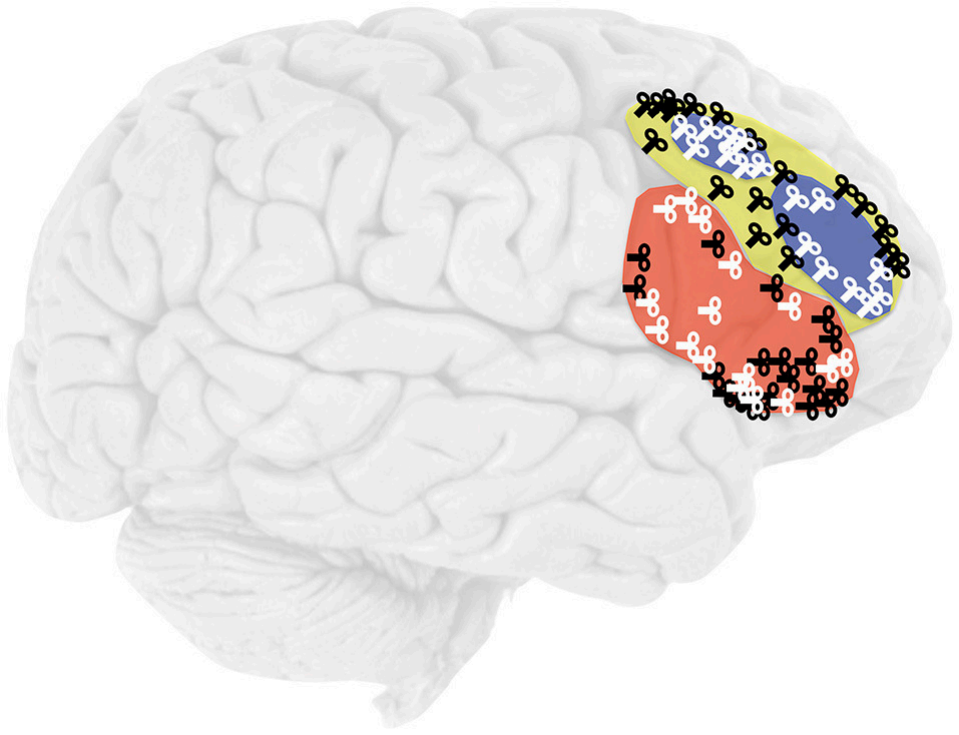
Location of cTBS in the rIFG (red), rMFG (yellow), and rDLPFC (blue). The coils represent the subject-specific target sites for the 20 participants in Experiment 1 (black) and the 16 participants in Experiment 2 (white).

Group-level (*N* = 36) whole-brain fMRI analyses of the H > CR contrast yielded significant differential activity across widespread fronto-parietal cortices (Figure 3). These results are consistent with the results of Aminoff et al. (2015) for participants who maintained a conservative decision criterion during recognition memory of faces (Table 1). The individualized cTBS target sites derived from subject-level fMRI analyses of the H > CR contrast are depicted in Figure 4.

**Table 1:** fMRI local maxima for Hits > Correct Rejections when participants (*N* = 36) maintained a conservative criterion during recognition memory tests (see also Figure 3).

**Cluster**	**Voxels**	***Z*-value**	**X**	**Y**	**Z**	**Location**	**BA**
1	7,748	5.32	−40	−66	−20	Left Cerebellar Lobule VI	19
1		5.12	40	−56	−28	Right Cerebellum crus I	37
1		5	36	−68	−16	Right Fusiform Gyrus	19
1		4.99	−36	−70	−20	Left Cerebellar Lobule VI	19
1		4.93	38	−54	−18	Right Fusiform Gyrus	37
1		4.92	34	−64	−24	Right Cerebellar Lobule VI	19
2	3,823	6.11	0	36	40	Left Medial Superior Frontal Gyrus	9
2		5.79	−4	26	32	Left Anterior Cingulate Cortex	24
2		5.69	0	30	46	Left Medial Superior Frontal Gyrus	8
2		5.33	−2	30	38	Left Medial Superior Frontal Gyrus	32
2		5.3	6	14	54	Right Supplementary Motor Area	6
2		5.27	−4	44	18	Left Anterior Cingulate Gyrus	32
3	3,468	6.3	32	20	6	Right Insula	48
3		5.32	30	30	2	Right Insula	47
3		5.14	42	10	28	Right Inferior Frontal Gyrus	44
3		4.96	44	12	−6	Right Insula	48
3		4.78	50	10	42	Right Precentral Gyrus	48
3		4.69	28	22	−10	Right Insula	47
4	1,990	5.4	−8	12	6	Left Caudate	25
4		5.02	−10	−2	20	Left Caudate	–
4		4.95	12	4	12	Right Caudate	–
4		4.89	−8	−2	8	Left Thalamus	–
4		4.69	10	14	12	Right Caudate	–
4		4.68	−10	4	14	Left Caudate	–
5	1,937	5.17	−28	−64	44	Left Inferior Parietal Lobule	7
5		5.09	−28	−52	42	Left Inferior Parietal Lobule	7
5		4.68	−36	−48	52	Left Inferior Parietal Lobule	40
5		4.52	−42	−40	52	Left Inferior Parietal Lobule	40
5		4.42	−42	−48	52	Left Inferior Parietal Lobule	40
5		4.39	−34	−56	52	Left Inferior Parietal Lobule	7
6	1,345	6.24	−34	16	4	Left Insula	48
6		5.85	−36	18	−2	Left Insula	47
6		5.84	−32	20	−2	Left Insula	47
6		5.56	−28	26	4	Left Insula	47
6		4.93	−38	12	−6	Left Insula	48
6		4.64	−48	16	−4	Left Inferior Frontal Gyrus	48
7	1221	5.79	34	−62	52	Right Superior Parietal Lobule	7
7		5.27	32	−64	38	Right Middle Occipital Gyrus	7
7		3.6	30	−72	32	Right Middle Occipital Gyrus	19
7		3.3	42	−44	54	Right Inferior Parietal Lobule	40
7		3.27	46	−44	56	Right Inferior Parietal Lobule	40
8	900	4.94	−48	22	32	Left Inferior Frontal Gyrus	44
8		4.45	−50	8	36	Left Precentral Gyrus	44
8		4.33	−42	24	24	Left Inferior Frontal Gyrus	48
8		3.92	−42	2	32	Left Precentral Gyrus	6
8		3.88	−44	8	28	Left Inferior Frontal Gyrus	44
8		3.82	−44	4	28	Left Precentral Gyrus	44
9	565	4.98	2	−32	28	Left Posterior Cingulate Gyrus	23
9		4.82	−4	−24	30	Left Midcingulate Area	23
9		4.7	−2	−18	32	Left Midcingulate Area	23
9		4.63	4	−16	32	Right Midcingulate Area	23
10	416	4.52	−46	46	2	Left Inferior Frontal Gyrus	45
10		4.29	−42	52	6	Left Middle Frontal Gyrus	46
10		4.29	−38	54	10	Left Middle Frontal Gyrus	46
10		4.29	−36	52	6	Left Middle Frontal Gyrus	10
10		4.09	−36	50	2	Left Middle Frontal Gyrus	47
10		4.08	−36	46	0	Left Middle Frontal Gyrus	47

*BA, Broadmann Area; X, Y, and Z coordinates are reported in MontrealNeurological Institute space*.

### cTBS effects on discriminability and criterion placement

**Figure 5:**
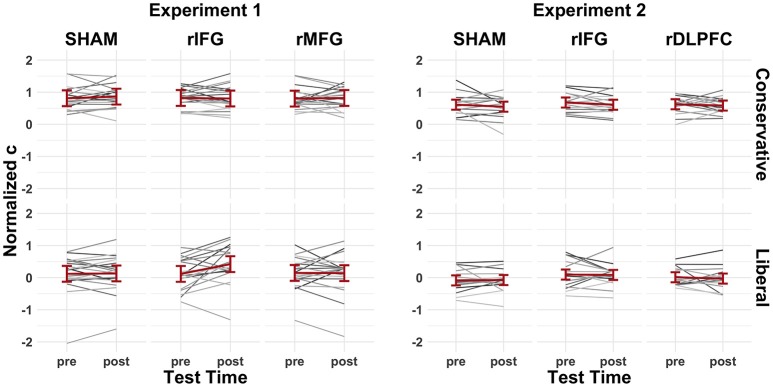
The pre-/post-cTBS normalized c values for Experiment 1 (left) and Experiment 2 (right). Gray lines indicate individual subject performance and red lines represent group averages fitted with 95% confidence intervals.

**Figure 6:**
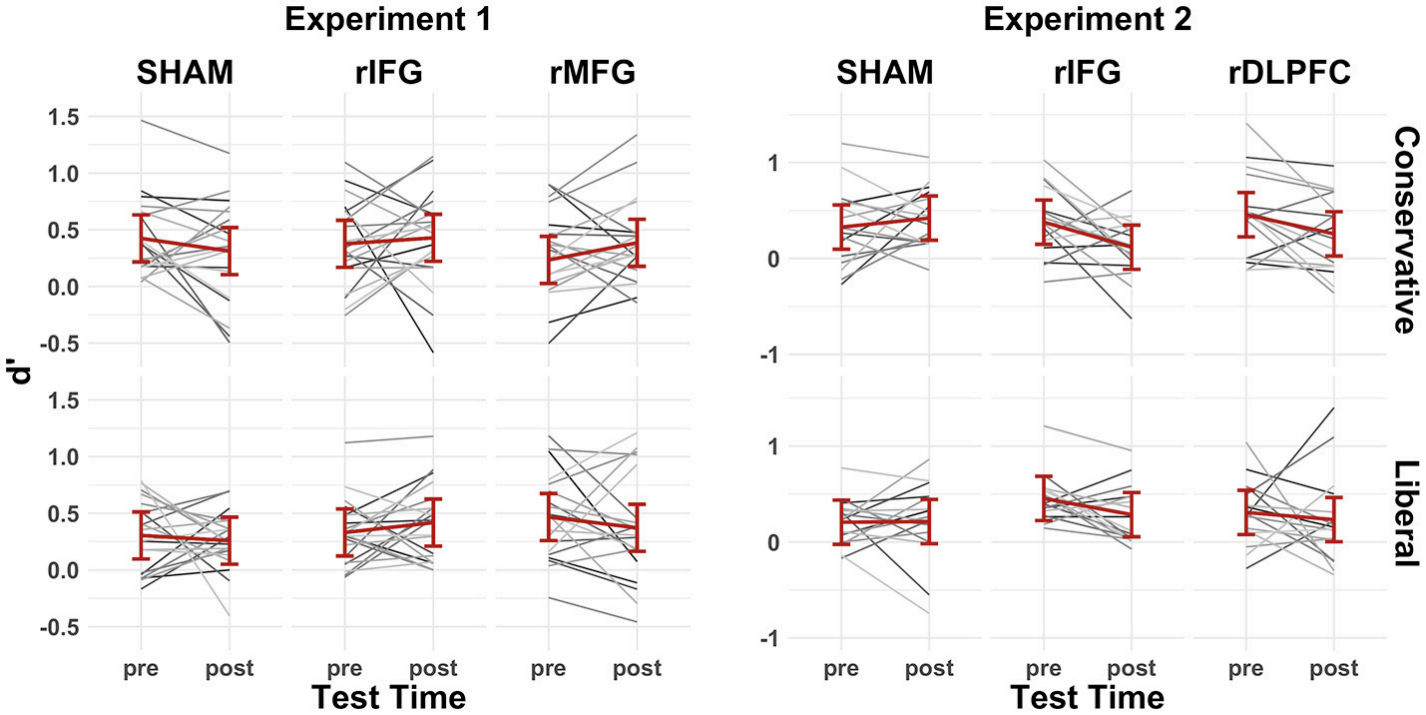
The pre-/post-cTBS'd values for Experiment 1 (left) and Experiment 2 (right). Gray lines indicate individual subject performance and red lines represent group averages fitted with 95% confidence intervals.

Average behavioral performance during the pre-cTBS memory tests in Experiment 1 revealed participants successfully shifted their decision criteria in response to the conservative (*c* = 0.81, *SD* = 0.32) and liberal (*c* = 0.13, *SD* = 0.53) probability manipulation (*p* < 0.001, *d* = 1.84) (Figure 5; *left*). Experiment 2 revealed similar results in the conservative (*c* = 0.64, *SD* = 0.30) and liberal (*c* = 0.01, *SD* = 0.36) criterion conditions (*p* < 0.001, *d* = 3.50) (Figure 5; *right*). Although participants on average maintained a slightly conservative bias in the liberal condition, the important distinction is that participants shifted to a *relatively* more liberal criterion. Mean discriminability remained low in Experiment 1 (*d'* = 0.36, *SD* = 0.37) and Experiment 2 (*d'* = 0.36, *SD* = 0.35) for the pre-cTBS memory tests, making it strategic to shift decision criteria (Figure 6).

#### Experiment 1

**Figure 7:**
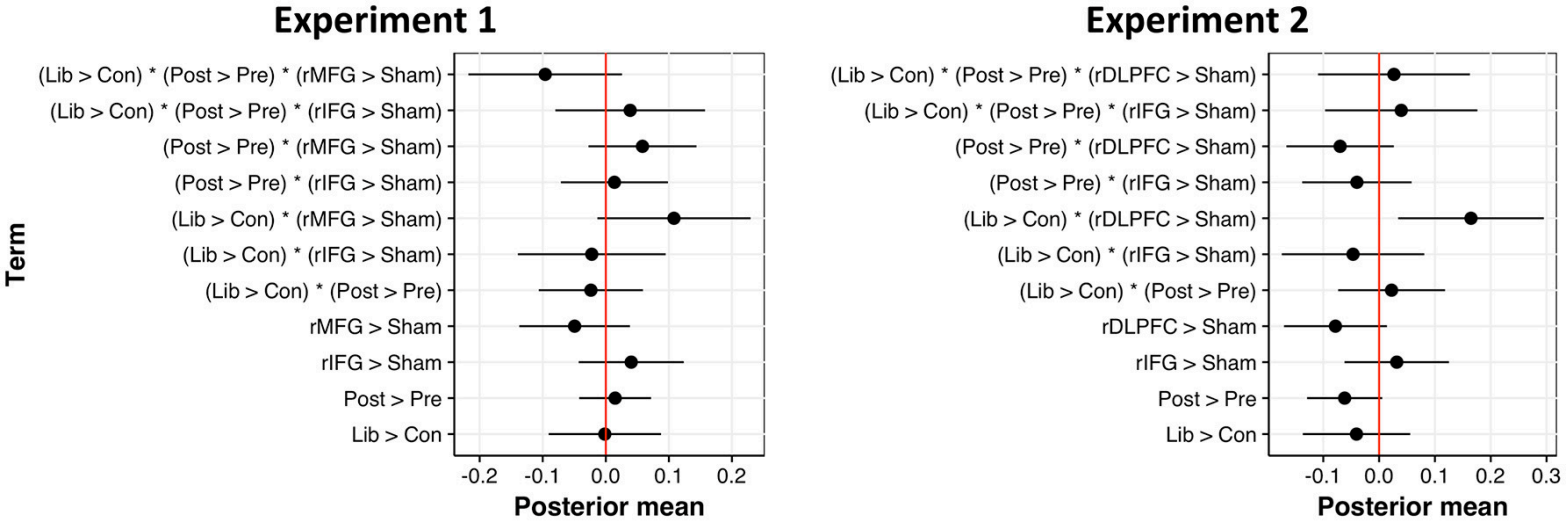
Posterior mean of parameter estimates across fixed effects for d' models, fitted with 95% confidence intervals for Experiment 1 (left) and Experiment 2 (right). Estimates not intersecting zero are statistically significant.

As predicted, applying cTBS to regions previously associated with criterion placement did not affect *d'*. The criterion manipulation also did not affect discriminability nor did performing the task pre- vs. post-cTBS. Figure 7 (*left*) displays the posterior mean parameter estimates and mean discriminability across factor levels for *d'* fitted with 95% confidence intervals; Table 2 (*left*) contains a summary of all model-level statistics.

**Table 2:** Model-level statistics ford' in Experiment 1 (left) and Experiment 2 (right).

**Term**	**Estimate (95CI)**	***SE***	***t***	**Effect size (*d*)**
**MODEL-LEVEL STATISTICS: D' (EXPERIMENT 1)**				
(Intercept)	0.36	0.052	6.95	0.942
	(0.256, 0.462)			
Lib > Con	−0.002	0.043	−0.043	0.005
	(−0.082, 0.081)			
Post > Pre	0.015	0.031	0.505	0.04
	(−0.047, 0.078)			
rIFG > Sham	0.043	0.043	0.991	0.111
	(−0.038, 0.128)			
rMFG > Sham	−0.05	0.043	−1.175	0.132
	(−0.137, 0.034)			
(Lib > Con) ^*^ (Post > Pre)	−0.024	0.043	−0.567	0.064
	(−0.114, 0.061)			
(Lib > Con) ^*^ (rIFG > Sham)	−0.027	0.061	−0.439	0.07
	(−0.146, 0.090)			
(Lib > Con) ^*^ (rMFG > Sham)	0.112	0.061	1.836	0.292
	(−0.015, 0.237)			
(Post > Pre) ^*^ (rIFG > Sham)	0.011	0.043	0.259	0.029
	(−0.071, 0.097)			
(Post > Pre) ^*^ (rMFG > Sham)	0.06	0.043	1.402	0.158
	(−0.022, 0.141)			
(Lib > Con) ^*^ (Post > Pre) ^*^ (rIFG > Sham)	0.042	0.061	0.683	0.109
	(−0.081, 0.159)			
(Lib > Con) ^*^ (Post > Pre) ^*^ (rMFG > Sham)	−0.099	0.061	−1.625	0.258
	(0.212, 0.013)			
**RANDOM EFFECT: (INTERCEPT | SUBJECT)**				
# of Subjects	20			
(Intercept) Standard Deviation	0.188			
*N*	240			
**MODEL-LEVEL STATISTICS: D' (EXPERIMENT 2)**				
(Intercept)	0.327	0.05	6.486	0.901
	(0.233, 0.421)			
Lib > Con	−0.043	0.048	−0.904	0.119
	(−0.136, 0.049)			
Post > Pre	−0.061	0.034	−1.814	0.169
	(−0.127, 0.007)			
rIFG > Sham	0.03	0.048	0.637	0.084
	(−0.062, 0.125)			
rDLPFC > Sham	−0.078	0.048	−1.641	0.216
	(−0.174, 0.016)			
(Lib > Con) ^*^ (Post > Pre)	0.022	0.048	0.454	0.06
	(−0.075, 0.117)			
(Lib > Con) ^*^ (rIFG > Sham)	−0.043	0.067	−0.638	0.119
	(−0.175, 0.086)			
(Lib > Con) ^*^ (rDLPFC > Sham)	0.165	0.067	2.44	0.454
	(0.033, 0.298)			
(Post > Pre) ^*^ (rIFG > Sham)	−0.039	0.048	−0.81	0.107
	(−0.132, 0.055)			
(Post > Pre) ^*^ (rDLPFC > Sham)	−0.07	0.048	−1.458	0.192
	(−0.161, 0.025)			
(Lib > Con) ^*^ (Post > Pre) ^*^ (rIFG > Sham)	0.041	0.067	0.604	0.112
	(−0.088, 0.172)			
(Lib > Con) ^*^ (Post > Pre) ^*^ (rDLPFC > Sham)	0.025	0.067	0.363	0.068
	(−0.110, 0.157)			
**RANDOM EFFECT: (INTERCEPT | SUBJECT)**				
# of Subjects	16			
(Intercept) Standard Deviation	0.15			
*N*	192			
				

**Figure 8:**
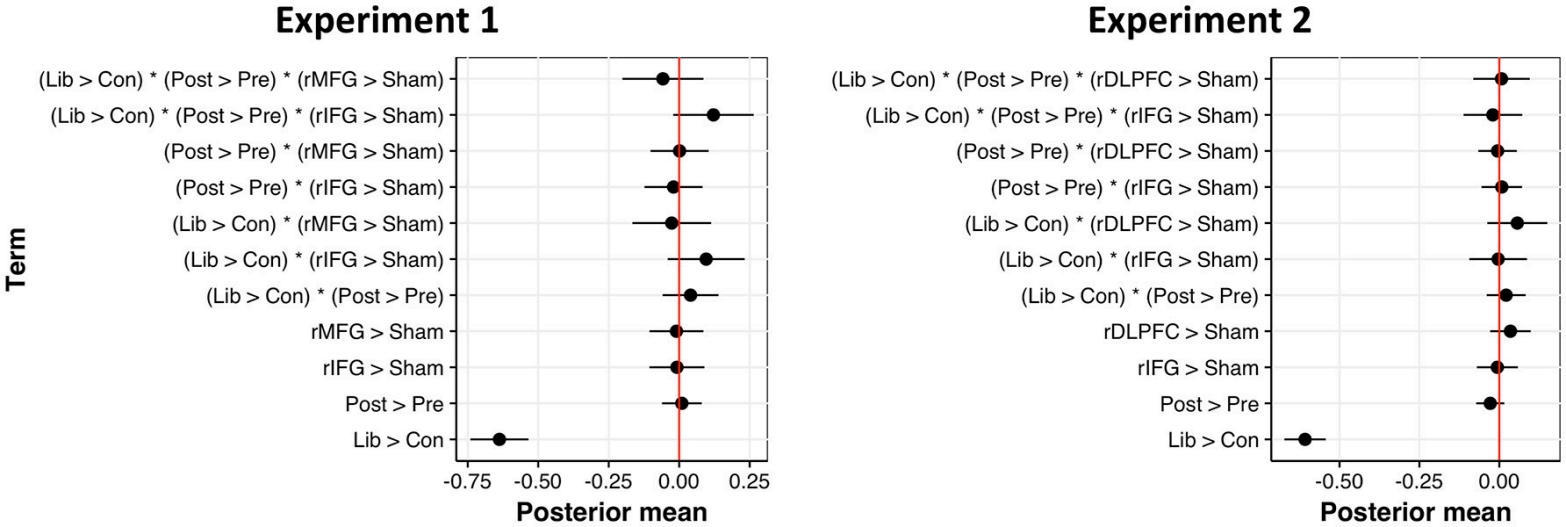
Posterior mean of parameter estimates across fixed effects for normalized c models, fitted with 95% confidence intervals for Experiment 1 (left) and Experiment 2 (right). Estimates not intersecting zero are statistically significant.

With respect to normalized *c*, we observed a significant main effect of criterion condition, such that participants set a more liberal decision criterion when target probability remained high (*b* = −0.64, 95CI = [−0.74, −0.54], *SE* = 0.05, *t* = −12.53, *d* = 1.37). Contrary to expectation, however, cTBS failed to affect one's criterion placement. Rather than *decreasing* the conservativeness of decision criteria, we found a marginal trend toward *more stringent* decision criteria following rIFG stimulation, as revealed by an interaction between criterion condition and cTBS to the rIFG, relative to sham (*b* = 0.10, 95CI = [−0.04, 0.24], *SE* = 0.07, *t* = 1.35, *d* = 0.21). A similar trend existed in the three-way interaction between our criterion manipulation, pre-/post-cTBS tests, and stimulation of the rIFG target (*b* = 0.12, 95CI [−0.02, 0.26], *SE* = 0.07, *t* = 1.63, *d* = 0.25). Summaries of model-level statistics are shown in Figure 8 (*left*) and Table 3 (*left*). This intriguing trend compelled us to collect more data to test whether the observed difference is truly a null result or merely an underpowered effect.

**Table 3:** Model–level statistics for normalized c in Experiment 1 (left) and Experiment 2 (right).

**Term**	**Estimate (95CI)**	***SE***	***t***	**Effect size (*d*)**
**MODEL–LEVEL STATISTICS: NORMALIZED C (EXPERIMENT 1)**				
(Intercept)	0.819	0.066	12.407	1.757
	(0.688, 0.954)			
Lib > Con	−0.639	0.051	−12.529	1.371
	(−0.738, −0.543)			
Post > Pre	0.009	0.036	0.249	0.019
	(−0.063, 0.080)			
rIFG > Sham	−0.008	0.051	−0.151	0.017
	(−0.107, 0.092)			
rMFG > Sham	−0.009	0.051	−0.186	0.02
	(−0.112, 0.091)			
(Lib > Con) ^*^ (Post > Pre)	0.042	0.051	0.829	0.091
	(−0.053, 0.141)			
(Lib > Con) ^*^ (rIFG > Sham)	0.097	0.072	1.35	0.209
	(−0.046, 0.243)			
(Lib > Con)^*^ (rMFG > Sham)	−0.026	0.072	−0.365	0.056
	(−0.167, 0.121)			
(Post > Pre) ^*^ (rIFG > Sham)	−0.017	0.051	−0.338	0.037
	(−0.117, 0.081)			
(Post > Pre) ^*^ (rMFG > Sham)	0.001	0.051	0.017	0.002
	(−0.100, 0.099)			
(Lib > Con) ^*^ (Post > Pre) ^*^ (rIFG > Sham)	0.118	0.072	1.633	0.253
	(−0.013, 0.253)			
(Lib > Con) ^*^ (Post > Pre) ^*^ (rMFG > Sham)	−0.056	0.072	−0.777	0.12
	(−0.192, 0.081)			
**RANDOM EFFECT: (INTERCEPT | SUBJECT)**				
# of Subjects	20			
(Intercept) Standard Deviation	0.247			
*N*	240			
**MODEL–LEVEL STATISTICS: NORMALIZED C (EXPERIMENT 2)**				
(Intercept)	0.608	0.064	9.489	1.854
	(0.477, 0.736)			
Lib > Con	−0.608	0.032	−18.776	1.852
	(−0.672, −0.542)			
Post > Pre	−0.029	0.023	−1.252	0.087
	(−0.073, 0.018)			
rIFG > Sham	−0.005	0.032	−0.158	0.016
	(−0.067, 0.06)			
rDLPFC > Sham	0.034	0.032	1.066	0.105
	(−0.030, 0.095)			
(Lib > Con) ^*^ (Post > Pre)	0.022	0.032	0.679	0.067
	(−0.042, 0.083)			
(Lib > Con) ^*^ (rIFG > Sham)	−0.004	0.046	−0.083	0.012
	(−0.094, 0.084)			
(Lib > Con) ^*^ (rDLPFC > Sham)	0.056	0.046	1.215	0.169
	(−0.032, 0.146)			
(Post > Pre) ^*^ (rIFG > Sham)	0.008	0.032	0.263	0.026
	(−0.060, 0.073)			
(Post > Pre) ^*^ (rDLPFC > Sham)	−0.006	0.032	−0.185	0.018
	(−0.070, 0.060)			
(Lib > Con) ^*^ (Post > Pre) ^*^ (rIFG > Sham)	−0.022	0.046	−0.486	0.068
	(−0.111, 0.069)			
(Lib > Con) ^*^ (Post > Pre) ^*^ (rDLPFC > Sham)	0.007	0.046	0.161	0.022
	(−0.083, 0.096)			
**RANDOM EFFECT: (INTERCEPT | SUBJECT)**				
# of Subjects	16			
(Intercept) Standard Deviation	0.24			
*N*	192			

#### Experiment 2

Since cTBS to the rMFG proved completely ineffective at affecting decision criteria, we switched our anatomically-defined rMFG ROI to a functionally-defined rDLPFC ROI for subsequent data collection (in case the broad rMFG ROI encompassed brain areas unrelated to maintaining a conservative criterion). Analyses on an additional 16 participants also revealed no significant interactions between task time, cTBS target site (rIFG > sham, rDLPFC > sham), and criterion condition. Results from Experiment 2 suggest that the trending interaction of pre-/post-cTBS, criterion condition, and rIFG stimulation (relative to sham) in Experiment 1 is likely a true null result (*b* = −0.02, 95CI = [−0.11, 0.07], *SE* = 0.05, *t* = −0.49, *d* = 0.07). Figure 8 (*right*) displays the posterior mean parameter estimates across factor levels for normalized *c* fitted with 95% confidence intervals; Table 3 (*right*) contains a summary of all model-level statistics.

Although we predicted no differences in discriminability, a significant interaction emerged between cTBS target site and criterion condition (Figure 7 (*right*); Table 2 (*right*)). Relative to sham, cTBS to the rDLPFC improved *d'* performance—specifically in the liberal condition (*v* = 0.17, 95CI—[0.03, 0.30, *SE* = 0.07, *t* = 2.44, *d* = 0.45). Although this is merely a two-way interaction (i.e. is agnostic to pre-/post-stimulation differences), it is nevertheless a moderately strong effect, and it raises the intriguing possibility that changing our target site from the rMFG to a more localized rDLPFC region directly affected a recognition memory network.

## Discussion

This study attempted to further illuminate neural mechanisms underlying the maintenance of a conservative decision criterion during recognition memory. Patients with damaged and/or dysfunction frontal lobes oftentimes establish overly liberal decision criteria when making recognition judgments (Biesbroek et al., 2015; Deason et al., 2017). In healthy individuals, widespread fronto-parietal BOLD activity is present in the H > CR contrast of recognition memory tests when maintaining a conservative decision criterion, but not a liberal criterion (Aminoff et al., 2015). These findings suggest that a conservative criterion may require an intact and functional prefrontal cortex. In particular, we investigated whether regions involved in response inhibition, such as the rIFG (Aron et al., 2014), mediate a conservative criterion by suppressing one's tendency to classify familiar items as old. We tested whether cTBS to the rIFG, rMFG, and rDLPFC (regions where increased BOLD activity tracks with the conservativeness of a decision criterion; Aminoff et al., 2015) causes participants to establish less conservative decision criteria during recognition memory. Participants initially conducted a recognition memory test while maintaining a conservative decision criterion during fMRI scanning. Despite obtaining subject-specific target sites based on peak BOLD activity in the H > CR contrast and using high-definition TMS equipment, cTBS to these sites did not significantly affect criterion placement.

There are several possible reasons why cTBS did not cause participants to establish less conservative decision criteria during recognition memory tests. First, our hypothesis that the rIFG, rMFG, and rDLPFC are *necessary* for maintaining a conservative decision criterion could simply be incorrect. Many studies investigating the neural substrates of criterion placement are correlational; thus, there may not be a direct causal relationship between our targeted regions and the maintenance of a conservative decision criterion. However, it would be difficult to reconcile this conclusion with results from studies showing that frontal lobe damage is commonly associated with more liberal decision criteria (but see Verfaellie et al., 2004; Hwang et al., 2007). Another possibility is that we did not target the appropriate regions within the rIFG, rMFG, and rDLPFC. We obtained subject-specific target sites via the H > CR contrast from a relatively short fMRI scanning session, which may not have provided the precise target sites of neural hubs that drive the maintenance of a conservative decision criterion. Future studies should consider obtaining subject-specific target sites via functional connectivity analyses from longer scanning sessions. There are also inherent technical difficulties with TMS that may explain our null findings, even *if* we targeted the correct brain regions. Sandrini et al. (2011) outline several technical considerations that affect the efficacy of TMS, including the type of stimulation protocol, intensity of stimulation, and the orientation of the coil handle.

Even if the most robust TMS protocol and coil positioning technique is employed, individual differences in anatomy and cortical excitability may cause wide variability in behavioral changes across participants. For instance, the efficacy of cTBS on inhibiting the primary motor cortex is quite variable (Suppa et al., 2016) despite being one of the few brain regions that give a measurable output via motor evoked potentials. The frontal cortex is also highly interconnected, which may contribute to more inter-individual variability in the effects of cTBS. For instance, Lee and D'Esposito (2012) observed that individuals with greater functional connectivity between the left and right IFG tended to have less of a decrement in working memory performance following cTBS to the left IFG. The authors suggested that the right IFG might play a compensatory role that reduces the behavioral detriments caused by left IFG inhibition. It is possible that inhibiting a small region within the right prefrontal cortex is easily compensated for since maintaining a conservative criterion may involve a widespread bilateral fronto-parietal network (Aminoff et al., 2015). Lastly, our participants conducted the recognition memory task several times, including prescreen and MRI sessions. It is possible that performing the task multiple times allow participants to develop efficient strategies that make it more difficult to disrupt behavior with cTBS. Nevertheless, due to the vast procedural parameter space that may vary the efficacy of neurostimulation, it is inappropriate to conclude that the rIFG, rMFG, and rDFLPC are *unnecessary* for maintaining a conservative decision criterion during recognition memory. Rather, it may simply be the particular cTBS technique itself that failed to affect decision criteria (whether or not the targeted regions are indeed implicated in maintaining a decision criterion), and we caution against its use in future investigations of the neural mechanisms underlying decision criteria.

Surprisingly, we did observe a significant interaction where participants improved *d'* performance specifically when maintaining a liberal criterion following cTBS to the rDLPFC. This finding is difficult to interpret because criterion placement should not influence discriminability (Macmillan and Creelman, 2005). Thus, an improvement in *d'* should be observed in *both* the conservative and liberal criterion conditions if cTBS indeed manipulated the discriminability of items at test. Due to our small sample size (*N* = 16) and the unpredicted nature of this finding, we hope to further investigate the robustness of this effect. If this finding is upheld with future research, it adds more complexity to the debate over whether the H > CR contrast is a function of retrieval itself (i.e. memory strength) or an epiphenomenal process such as criterion setting.

Although the present investigations failed to manipulate criterion placement with cTBS, other TMS methods may ultimately prove more successful. We suggest future studies employ *online* TMS protocols to ensure target sites are being stimulated *while* participants perform recognition memory tasks with decision criterion manipulations. We employed an offline cTBS approach in hopes of conducting a future study in which participants perform post-stimulation recognition tests during fMRI scanning. However, our null results suggest that cTBS to the rIFG, rMFG, and rDLPFC are ineffective at manipulating criterion placement during a recognition memory test.

## Conclusion

Offline cTBS to the rIFG, rMFG, and rDLPFC proved ineffective at altering decision criteria during recognition memory tests. This is not to suggest that these frontal regions are uninvolved in the maintenance of a conservative decision criterion or that TMS generally cannot affect criterion placement. However, we do not recommend using offline cTBS to manipulate decision criteria during recognition memory. An unexpected finding of increased memory accuracy (*d'*) when maintaining a liberal criterion following cTBS to the rDLPFC could motivate future research investigating prefrontal neural networks involved in recognition memory.

## Data availability

Datasets for this study can be accessed through the Open Science Framework: https://osf.io/r73xg/.

## Author contributions

EL designed the study, conducted the experiment, performed data analysis and wrote the manuscript. TS helped performed data analysis and edited the manuscript. LV helped design the study. MM helped design the study, edited the manuscript and oversaw all research activities.

### Conflict of interest statement

The authors declare that the research was conducted in the absence of any commercial or financial relationships that could be construed as a potential conflict of interest. The reviewer KD and handling Editor declared their shared affiliation.
